# An examination of pharmaceutical systems in severely disrupted countries

**DOI:** 10.1186/1472-698X-12-34

**Published:** 2012-12-06

**Authors:** Jillian Clare Kohler, Enrico Pavignani, Markus Michael, Natalia Ovtcharenko, Maurizio Murru, Peter S Hill

**Affiliations:** 1 Associate Professor, Leslie Dan Faculty of Pharmacy and Munk School of Global Affairs, University of Toronto, Toronto, Canada; 2 Lecturer, School of Population Health, University of Queensland, Brisbane, Australia; 3 Consultant for Public Health and Humanitarian Aid, Sao Paulo, Brazil; 4 Research Assistant, Initiative for Drug Equity and Access, University of Toronto, Toronto, Canada; 5 Public Health Consultant, Modena, Italy; 6 Associate Professor, School of Population Health, University of Queensland, Brisbane, Australia

## Abstract

This research assesses informal markets that dominate pharmaceutical systems in severely disrupted countries and identifies areas for further investigation. Findings are based on recent academic papers, policy and grey literature, and field studies in Somalia, Afghanistan, the Democratic Republic of Congo and Haiti. The public sector in the studied countries is characterized in part by weak Ministries of Health and low donor coordination. Informal markets, where medicines are regularly sold in market stalls and unregulated pharmacies, often accompanied by unqualified medical advice, have proliferated. Counterfeit and sub-standard medicines trade networks have also developed. To help increase medicine availability for citizens, informal markets should be integrated into existing access to medicines initiatives.

## Introduction

Pharmaceuticals are indispensable to health systems; they complement other types of health care services to reduce morbidity and mortality rates and enhance quality of life. Pharmaceuticals are unlike ordinary commodities given their therapeutic properties; people rely on them intensely for health, sometimes even life itself. Pharmaceutical supply systems are a critical constraint for making progress in health outcomes within the spectrum of treatable diseases; they need to work well if health gains are going to be made. However, owing to pharmaceutical supply system shortcomings, the poor continue to die from treatable infectious diseases worldwide each year.

One of the State’s core roles in meeting essential needs is maintaining a functioning health system with an adequate supply of essential medicines, enabling its citizens to exercise their right to a standard of living that guarantees health and well-being as outlined in the Universal Declaration of Human Rights. As emphasized by Kohler et al., “[r]egulation of health sector goods and services is a core function of government. Governments are responsible for ensuring that health professionals are licensed properly and that health products are safe and effective” [1]. The ability of states to do so varies throughout developing countries given limited resources and oftentimes less than optimal healthcare infrastructure and human resources. It is of particular difficulty in severely disrupted states where governance is weak and intra-state conflict can be the status quo. What is often overlooked in many pharmaceutical systems is the importance of ensuring that good governance is in place. Governance is defined by the United Nations Development Programme (UNDP) as:

“(T)he exercise of economic, political, and administrative authority to manage a country’s affairs at all levels, comprising the mechanisms, processes, and institutions through which that authority is directed. Good governance is, among other things, participatory, transparent, accountable, and efficient. It promotes the rule of law and equal justice under the law. Governance requires the involvement of the private sector, civil society and the state and is a prerequisite for sustainable human development” [2].

The implementation of good governance requires a functioning state. We seek to explore what happens to governance within the context of the pharmaceutical system when states break down or fail. Definitions of what characterizes a failed state are diverse but include longstanding entrenched weaknesses. For example, the Organization for Economic Cooperation and Development (OECD) definition includes an inability for a state to sustain itself over time and Robert Zoellick describes the persistence of poverty as a consequence of overlapping weaknesses between governance, poverty and conflict [3,4]. This however does not encompass the entire definition of what a failed state is. Failed states often have a regional significance due to their negative externalities (i.e. for smuggling and trade of counterfeit medicines) and share common problems such as poverty, insecurity, and proneness to conflict [5,6]. Given the debate surrounding the term ‘failed state, ’ the term ‘severely disrupted country’ will be used instead to describe the countries under study. This term also conveys the state of affairs in the countries under study where state administrations, public health systems or pharmaceutical systems function weakly, if at all.

Informal mechanisms in these instances are not surprisingly resilient. In the absence of governance or with weak governance, pluralistic health systems made up of a collection of public and private health care providers operate with little communication amongst each other [7]. Walle and Ponsaers define informal markets as those without state intervention [8]. Because they are outside of the purview of state regulation, these markets also do not necessarily entail ‘illegal economic activity’. When public health systems fail to provide pharmaceuticals to patients effectively, citizens will turn even more intensely to private (formal and informal) providers and are often unaware of the possible risks involved or despite those risks. It is critical to bear in mind that there are no universal demarcations between public, private, and informal markets. For example, there may be formal and informal dispensing of medicines taking place in public facilities and in the private market.

Unlike unstable governments, which can fracture at any time, the states being studied are “chronically stable” in their inability to service their population’s needs. The institutions, though largely ineffective, have been in place for many years and are unlikely to change. Well regulated institutions are usually considered to be essential to the pharmaceutical sector where medicines are procured in large amounts and must then be checked to ensure quality, stored properly, distributed, and dispensed based on accurate diagnoses of health issues. As it is difficult to imagine significant change in the institutional structure of the states being examined in the near future, they must be examined from a different perspective. A paradigm shift is necessary to understand how the provision of pharmaceuticals may be made safer and more transparent.

This paper illuminates how informal markets flourish in severely disrupted countries. In the absence of sufficient public health systems, patients are opportunistic and turn to where drug products are available - even though the quality and efficacy of these products may be in question. It is difficult to draw generalizations from the country examples we will highlight in our paper given the unique circumstances of each country. What we aim to demonstrate, however, is the importance of informal markets in difficult country conditions and the need for policy makers to address access to medicines in a pragmatic way by working with existing conditions, however imperfect.

We organize our article as follows: first, we present our methods. Second, we use case studies: Afghanistan, Somalia, Haiti, and the Democratic Republic of Congo (DRC) to illustrate some of the major issues facing pharmaceutical supply in severely disrupted countries. Each case study will characterize the public and informal sector as pertaining to pharmaceutical supply and their relation to the rational use of medicines and the trade and sale of counterfeit/substandard medicines. The discussion will then compare and contrast the case studies, drawing out key trends and proposing possible interventions and areas where further research is recommended. In this context, it is compelling to know how informal and spontaneous healthcare provision –namely pharmaceutical supply - has adapted to state degeneration and the subsequent benefits or dangers of such adaptation.

## Methods

Field studies were carried out in Afghanistan, Haiti, Somalia, the DRC, Palestine and the Central African Republic (CAF) as part of the research programme “Providing Health Care in Severely Disrupted Environments: A Multi-Country Study” at the University of Queensland. Although healthcare systems were examined as part of the programme, this paper focuses on the pharmaceutical sector, the results of the following case studies were the primary product of the research program: *Too good to be true? An assessment of health system progress in Afghanistan 2002–2010* (Markus Michael, 2011, unpublished manuscript), *The Somali healthcare arena. A (still incomplete) mosaic* (Enrico Pavignani, 2012, unpublished manuscript), *“Résultats périssables”: An assessment of the health system in Haïti* (Peter S. Hill and Markus Michael, 2012, unpublished manuscript), and *Democratic Republic of Congo. The chronically-ill heart of Africa. Draft* (Maurizio Murru and Enrico Pavignani, 2012, unpublished manuscript). Results from Palestine were not included within the scope of this study because its environment is significantly different from those covered in this paper, more over-governed than under-governed. The results from the CAF were not included as research was not complete during the preparation of this paper.

Key informant interviews were conducted for all countries studied. Interviews for the Somalia case study took place in the neighboring Nairobi and in Kinshasa, Goma and Kindu for the DRC. In Haiti, we conducted 45 key informant interviews in January 2011. Key informants were identified in consultation with professionals currently engaged in development in Haïti, with subsequent snowballing; a seminar convened by the *Université Notre Dame d’Haïti* provided direct contact with the local public health and academic communities, and access to the mailing lists for coordination of health services allowed identification of key humanitarian and development agencies. In Afghanistan, 41 interviews were conducted between the 26^th^ of November and 20^th^ of December 2010. The sample of respondents for Haiti and Afghanistan included representatives from the Ministry of Health, donors, NGOs, other humanitarian organizations, and UN agencies. Individual health professionals were interviewed while in Afghanistan. The researcher (M. Michael) draws on combined three years of experience with health care provision in Afghanistan.

All field studies included a literature search as well as document and policy analysis. Substantial efforts were devoted to finding, analyzing and triangulating as many documents as possible. The final reports relied to a greater degree on existing documentation than on interviews, as interviews often gave little new information on medicines. In contrast, emailing contacts gave researchers access to fairly unknown key documents. This was an important methodological lesson – valuable documentation is often unknown or forgotten by involved actors, but it does exist.

Because of time, financial and logistical constraints, it was not possible to expand coverage in the DRC and interview a greater number of people directly involved in the delivery of health services at local level. Among the main limitations of the Haiti study were the reliance on relatively short fieldwork and on secondary information sources to construct insights into a complex health sector. In Afghanistan, security concerns precluded the visit to more insecure provinces, as the researcher was not attached to any operational organization. Another limitation in Afghanistan was the lack of interviews involving representatives from the central level of donor institutions. We acknowledge that the choice of respondents may have introduced a provider bias.

## Findings

### Country case studies

#### Afghanistan

Afghanistan’s public health system is in large part a joint effort of the Ministry of Public Health (MOPH), donors, and not-for-profits. The private health sector ranges from qualified health professionals (often individuals who are also public providers) providing curative care to individuals with no technical skills or background knowledge selling medicines [9,10]. The level of regulation over the private sector varies wherein private pharmacies and grocery store vendors may be considered to be formal and market drug sellers informal.

Afghanistan’s health system has gone through extensive reforms since 2002, although most reforms have largely neglected the pharmaceutical area as well as other support systems. Up until that time, “the prolonged civil war, the shortage of staff in rural areas, and the absence of explicitly articulated national priorities all resulted in limited availability and quality of services” [9]. Since then, the MOPH has gone through a major restructuring. It has focused on providing a Basic Package of Health Services (BPHS) and Essential Package of Hospital Services (EPHS), primarily through contracting out to non-governmental organizations (NGOs) and international organizations. Some studies and public discourse suggest that the state’s efforts have been reasonably successful. On the other hand, key informants pointed out that the system is fragmented, there is a distinct lack of coordination between donors and the ministry, and it is unclear where the accountability lies for meeting goals [9-11]. Low coordination extends to the public pharmaceutical sector. There is no national supply system, nor is there one specific to the BPHS and EPHS (except for components led by USAID, which has outsourced to Tech Serve International).

There are three categories of vendors from whom citizens may purchase medicines: private pharmacies, grocery stores and street vendors. Jointly they provide at least 70% of the essential medicines in the country [12]. Private pharmacies are registered and subject to rules, such as the requirement that a pharmacist be on site at all times [13]. But this is not consistently implemented, as is commonly the case in many low and middle-income countries [13]. Grocery store vendors are the second level of provision and are also subject to reviews but street vendors are not regulated thereby falling into the informal category [13]. Cost may be a factor in the large number of street vendors as out of pocket expenditures account for 69% to 79% of health financing according to one survey [14]. Quantitative studies have found that citizens either preferred the private sector or rated it equally with the public when receiving care, and the prevalence of the pharmacies makes them a prominent option for purchasing medicines [15,14,10].

Regulatory controls are not actively implemented and the pharmaceutical sector as a whole is “understudied”, ‘highly complex and chaotic’ and ‘completely out of control in all respects’ [12]. Formally, quality control measures are reasonably established. Procedures are in place to send imported products to labs for quality checking and MOPH registration as needed. Yet even the government admits that the quality of pharmaceuticals imported and sold is not ensured [16]. With the most advanced public health coverage—though with disappointingly limited utilisation—it has yet to be possible to maintain security on the border and assess the quality of both registered and non-registered imports. Afghanistan’s porous and non-secure border connects it to several countries and allows multiple points of entry for medicines. The largest supplier is Pakistan, followed by Iran, India, China, the Middle East, and Europe [13]. Pakistan may have producers specifically targeting the Afghan market with their pharmaceutical supply [13]. Medicines transported across these borders include both branded and generics products as well as counterfeits and sub-standard pharmaceuticals. In addition to imports across Iranian and Pakistani borders directly, medicines from Iran can go through Turkmenistan and those from Central Asia go through Uzbekistan and Tajikistan [13]. Although legitimate trade in medicines is required for quality standard testing in Kabul, no screening is possible for those transported illegally.

Afghanistan’s programs cannot stop the trade of counterfeit medicines. This is true in the public sector, while in the private sector regulation is likely to be even less effective. More structured systems possibly lead to more structured pharmaceutical crime. For example, key informants suggested that counterfeit pharmaceuticals are made specifically for the Afghan market, which one study called a ‘pharmaceutical dustbin’ [12].

#### Somalia

Somalia’s public health facilities can be seen as those connected to zonal health authorities, often receiving financing from Western aid agencies. One author has suggested that the public-private distinction is meaningless and should not be used in the Somali context [17]. Public facilities are ostensibly bound to health authority policies but are not provided financing, requiring the use of user fees, nor overseen by them as expected [18,19]. The ‘public’ classification is therefore misleading as its actions are commercial, with health workers acting as sellers of merchandise. Other sources of healthcare can include providers funded by Islamic charities, traditional healers, and individually operated pharmacies. There are laws governing private health facilities in Somaliland, including pharmacies, but most private operators are unaware of them and they are not strongly enforced [20]. Consequently, there ought to be a formal private sector but in reality it seems more informal.

Much of the variability in the quality and range of health services available is due to Somalia’s unstable political situation. The three zonal Ministries of Health (Ministry of Health of the Transitional Federal Government, Ministry of Health and Labor of Somaliland, and the Ministry of Health of Puntland) operate with varying (and overall limited) levels of influence over domestic operations. Bilateral donors and international NGOs are the most significant contributors to the formal health care system but aid is uncoordinated, without common long-term agendas. UNICEF is an important player in the provision of primary healthcare, with a history of supplying Mother-and-Child (MCH) Centers and Health Posts with ration kits. But even in this program, record-keeping and reporting of what is being supplied have only been implemented recently.

The Somali healthcare network is diverse, active and growing but perceptions by commentators and even those working in the country are not commensurated with this characterization. These inaccurate perceptions could be explained by the lack of understanding of the significant presence of market pharmaceutical vendors and other private pharmacies as sources of health care. In Somalia, the number of private pharmacies has been growing since the 1980s. They are not restricted to urban areas, though they are still largely concentrated there [21]. Although public facilities are kept track of, private pharmacies are not. One count of private clinics and pharmacies recorded 190 providers, whereas pharmacies alone likely number in the thousands. It is likely that the whole pharmaceutical market, private and donor-supported combined, represents one of the largest sectors of the economy in value, although no records have been kept. A study by Mazzilli et al. [20] in Somaliland found that at least seven major importing firms were supplying a competitive network of wholesalers that in turn were selling products with almost 800 private pharmacies operating in the country. The same study found that there are close relationships between the private and public operators. As in Afghanistan, many employees in pharmacies were also public employees, suggesting that the dual jobs may be coping mechanisms to ensure sufficient wages for livelihood.

Marchal [22] examined the Bakaaraha market in Mogadishu, which included a large section for medicines consisting of as much as 55 stores and 350 booths and operated without any regulatory controls and in the absence of technical skills among the medicine suppliers. Not surprisingly, the quality of the products purchased in the market was a source of concern and certain donor and not-for-profit funded health facilities did not purchase medicines there because of their dubious provenance.

One key characteristic of the Somali pharmaceutical sector is that customers tend to prefer private pharmacies. They have longer operating hours, the significant number dispersed throughout the country make them accessible to a large proportion of the population, and high user fees discourage the use of public facilities. *“Outlets were generally small operations, serving light client loads with small profit margins. To make any profit the pharmacies were extremely efficient in stock management and were open long hours on most days of the week”*[20]. Easy access to medicines through private pharmacies is in line with the tendency of citizens to first seek traditional care and then purchase medicines, all prior to visiting a health facility which is viewed as simply a place to get a prescription [23].

Somalia is critical for the pharmaceutical trade because its duty-free conditions have made it a major import channel for the Horn of Africa (Kenya and Ethiopia). There are linkages between the pharmaceutical market in Ethiopia and Somalia, but little is known about the routes which are taken or the amount of goods that cross the border. Drug supply routes are the least investigated segment of the health system in Somalia although a study did find that most medicines in the central Bakaarha market in Mogadishu came from Pakistan and India [20-22]. One informant explained that the opaque nature of the pharmaceutical system could in fact be deliberate to facilitate illegal business. The ease and impunity with which counterfeit, expired or repackaged medicines can enter into a large market has obvious appeal to criminal networks.

#### Haiti

The Pan-American Health Organization (PAHO) broke divided Haiti’s health system into four sectors: 1) public; 2) private not-for-profit (NGOs and religious organizations); 3) mixed not-for-profit (private management with staff paid by the MSPP); 4) private for-profit [24]. Yet, functionally, informant interviews suggested the following categorization for Haiti’s health system: 1) formal facilities, administered by the Ministry of Public Health and Population (MSPP - Ministère de la Santé Publique et de la Population), NGOs, or privately; 2) informal facilities and practitioners, not registered or recognized by the MSPP and functioning outside the system; 3) individuals who play roles across these boundaries.

Lack of coordination is one of the many challenges in Haiti, likely one of the results of a weak health system. The earthquake has further disturbed the tenuous equilibrium of the public health system, though any major stressor would trigger a crisis in the fragile state. One of the outcomes was drastic increase in the number of NGO services operating independently of national public and private endeavors, though there have been early initiatives to coordinate the acute responses to cholera under the MSPP, and to extend this to other health issues. The MSPP has attempted to decentralize its operations, but although work areas are divided amongst multiple departments, financial and decision-making autonomy has not devolved with them [25,26]. It has also in large part failed to regulate other sectors, particularly NGOs. The MSPP directly controls only 10-15% of health services while NGOs manage most without coordination. NGOs may run facilities with salaries paid by the MSPP or they may support the MSPP in paying the salaries to its employees in a facility. In addition, NGOs may start their own facilities without going through the process of MSPP authorization.

Around 40 agencies import pharmaceuticals for both public and private markets in addition to the availability of central stores (Program on Essential Medicine and Supplies - PROMESS) in the public sector. This situation, along with the direct influx of pharmaceuticals through international actors, has limited government’s capacity to track the amount of medicines being distributed in the country, making it difficult to accurately project needs. The tension between the MSPP and NGOs is apparent in the recent attempt by the government to regulate imports. Registration policies have resulted in document processing delays of as long as six to nine months. Even though this presents some administrative delays for NGOs, it does help manage the quality of drugs at an early stage due to a lack of centralized distribution channels and poor quality drugs being able to enter through NGO supply channels as well as informal ones [25]. Furthermore, the new registration policies are meant to help avoid duplication and facilitate coordination. This can only work if NGOs cooperate and supply through the government’s procedures and if the government manages the scheme competently and honestly. Independent supply lines, however allow organizations to avoid them. A committee has also been set up with a long term aim of coordination but it remains in the early stages and focused on information sharing.

Unfortunately, the regulation of imports has not extended to street vendors, who are part of the informal sector (according to the second categorization of Haiti’s health services). These vendors “échappe à tout contrôle” (avoid all efforts of control) [27]. Street vendors and unregistered pharmaceutical sellers offer a range of antibiotics and popular analgesics. In addition, traditional medicine is recognized, even by senior health authorities, as the one health system “with 100% coverage”. Numerous factors inform individuals’ decisions to seek traditional health care, but limited access to health services and products is a particularly strong reason. In Artibonite there were only 2.7 health professionals for every 10,000 inhabitants. According to another study, 60% of the poorest households do not seek care due to costs [28]. Patients are responsible for the costs of medicines as well as other supplies when seeking procedures at public hospitals [29].

Policies aimed to curb the influx of counterfeit and substandard medicines are still in the early stages in Haiti. Haiti’s public sector primarily seeks procurement through the World Health Organization (WHO) supported PROMESS where at least some quality control measures are in place. Still, local purchases are at times necessary and local production is not well developed, not regulated, and not subject to quality control [30][25]. Even imported pharmaceuticals (both through public and NGO channels) are not of guaranteed quality, though as mentioned earlier, importers and eligible pharmaceuticals must now be registered, which is a positive development [30,25].

#### DRC

The DRC has a significantly decentralized public health system that includes many public-private partnerships [31]. With regards to the private sector, the 2005 World Bank Health, Nutrition and Population status report mentioned three different categories: private pharmacies, medicine sellers and informal healers [31]. Private pharmacies are headed by registered pharmacists and should be licensed, although the sheer number of private pharmacies makes this difficult to enforce [31]. As a result, though medicine sellers and healers are clearly more informal, the place of private pharmacies is not as evident.

The public health sector is decentralized on the one hand, with 515 Health Zones, but with a central reporting structure to the Ministry of Health [31]. In practice, it is difficult to see how regulation can be enforced by the Ministry of Health, which is extremely fragmented itself (13 Directorates and 52 Special Programmes at the central level, 11 Provincial Health Directorates and 65 Health Districts at the mid-level). The number of Health Zones was officially increased from 306 to 515 in 2001, but without the resources to support them, many old and new Zones are ghost structures and some were simply not put in place. Half of the country’s hospitals and health units are run by private institutions, churches and non-governmental organizations, “while the other half is state-run with no resources and personnel who have no equipment or medical supplies to work with. The public health system is still in “total collapse” [32].

Currently, the pharmaceutical supply chain is fragmented between private, public, and public-private groups [33]. A recent WHO study identified 19 procurement agencies with 52 partners, along with 99 distribution channels for different vertical programs [33]. The government set up the Système National d’Approvisionnement en Médicaments – National System for the Procurement of Medicines (SNAME) and the Programme National d’Approvisionnement en Médicaments – National Programme for the Procurement of Medicines (PNAM) in 2002. The system is founded on numerous autonomous not-for-profit agencies which include two procurement agencies and 15 local distribution stores. Though the concept is good, insufficient participation in SNAME limits its effectiveness and makes coverage of the health sector incomplete. While medicines in the public sector are procured through donors and NGOs a study which surveyed at least 39 private pharmacies found no connection to the public system in their procurement strategies; most used local suppliers [33,34]. The DRC facilities buy drugs from a variety of sellers, ranging from the professional to the plainly fraudulent. Their purchasing rationale is accordingly varied, from concern for the health of patients to the profit-maximizing one.

Both the private and informal sectors are important parts of the DRC pharmaceutical market. Informal business activities in general are integral to most citizens. The majority of citizens engage in some sort of informal activity, including 90% of Kinshasa’s population [35]. Surveys have found many private pharmacies in urban areas but even suburban and rural regions have drug shops [36]. Although they are not regulated and quality is questionable some surveys suggest almost a third of citizens prefer medicine sellers over “formal sector practitioners” [31]. A study of 39 pharmacies suggested that private pharmacies are not necessarily a safer choice; 42% did not have a pharmacist on the payroll and 38% had no prescription records [31]. In the past, the government did make an attempt to regulate the sale of pharmaceuticals. However, a strict approach was used which precluded the participation of informal sellers such as those in markets and consequently resulting little to change their practices [37].

As in all countries, numerous factors contribute to the choice of healthcare provider. Cost is a definite consideration, often leading to no treatment. The largest share of household expenditures (41%) goes to the purchase of medicines and high user fees for healthcare were shown to be a cause of underutilization – when the Health Zone of Rwanguba, North Kivu dropped user fees, utilization increased by 4.5 times [38,39]. But it is not as straightforward when types of providers are broken down. For example, drug sellers are more popular with the poor in urban areas while in rural areas they are more likely to be used by those who are better off [31]. The use of traditional medicine has not been quantified but is believed to be high [40]. The MSP quoted unreferenced surveys when noting that 70-80% of the population used traditional medicine [41]. A few small-scale surveys have found relatively low usage rates, but these low results are not in line with the received wisdom and trends in other countries [31]. Overall, there is a distinct lack of information on health-care seeking behavior and consequently the relative place of medicine sellers and other pharmaceutical actors within it.

Trans-border trade has not been examined in detail, but may account for a significant portion of the economy where it has been studied and likely exists in other areas as well [42]. The sale of DRC produced medicines was witnessed in Bangui, the capital of the CAR. The extent of the smuggling of pharmaceuticals out of the country is unknown but it does occur.

## Discussion

### Formal actors

The public sector of the health care system is where the involvement of donors, NGOs, and multilateral institutions is most evident. Arguably, donors are able to produce the most concrete outcomes in vertical, disease and location specific programs. As well, even in “normal” states there are ample challenges in human resources (both too low and too high), inefficient management, weak institutions, weak oversight and a lack of sufficient health infrastructure, to name a few. Severely disrupted countries present these challenges and more.

Multilateral institutions, other donors, and NGOs were important actors in all of the countries examined. They were an integral part of all of the health systems, either taking charge of facilities or providing care when government facilities could not. Lack of coordination among all of them was a key issue in all of the case studies. Efforts have been made to address this in some countries. For example, Somalia had a coordinating body for aid agencies but its effectiveness has declined over time. In Haiti, the lack of connection between aid agencies and the government is a concern; seeking authorization from the government is treated as a barrier rather than an indication of institutional development. Lack of coordination has resulted in the fragmentation of drug supply. One major area is the use of varied procurement techniques in the same country. All of these organizations form a critical part of the health system in severely disrupted countries and their constant presence is unlikely to change. The weak public health systems in each country have driven much of the public to other sources, such as private pharmacies and pharmaceutical vendors. Perception of low quality, low access, and high costs of use can result in use of these sources and lead to high-risk actions. The result of this transition is that individuals purchase pharmaceuticals without proper diagnoses and are at higher risk of obtaining counterfeit or sub-standard pharmaceuticals, raising the possibility of dangerous outcomes such as resistance to medications and death. These pharmaceuticals are also making individuals poorer without giving any health benefits.

### Informal supply

In the context of severely disrupted countries, we argue that it is not helpful to separate formal and informal markets completely: the relationship between the two markets is often symbiotic, with the systems supporting each other to varying degrees. But when dealing with pharmaceuticals there is a danger inherent to informal markets must be acknowledged. Improper pharmaceutical consumption can lead to pharmaceutical resistance and in the worst-case scenarios - death. The absence of enforceable regulation to avoid such outcomes is not necessarily limited to the informal markets; often there are insufficient resources to manage the supply of medicines in the public sector, putting the patient at risk.

Better understanding about the informal markets is needed among health care providers and donors. Further investigation to understand both their approximate numbers and distribution is critical to better understanding the role of these providers.

The framework of formal and informal markets is ultimately very limiting in examining the healthcare and pharmaceutical system. Each case study has a unique breakdown of service providers. At times there was a distinct public/private division on paper (Afghanistan and Haiti), but it was less clear in Somalia and the DRC. In practice the distinctions are blurred in all contexts, the difference being the increased visibility of the phenomenon in Somalia and the DRC. The formal/informal distinction is even more difficult to make. Regulations exist on paper in all countries but they are routineley ignored and when enforced they tend to be harmful. Basing the division on at least some type of enforcement being in place, in Afghanistan and possibly the DRC, formal and informal providers in the private sector could be distinguished. In contrast, basically all providers were informal in Haiti and Somalia. The area of informal sale of pharmaceuticals in the public sector was not covered in these case studies due to limits in available information but the category cannot be ignored and warrants greater research.

Traditional medicine was part of the health sector in each country as well. When seeking to understand informal healthcare providers, healers and other traditional medicine practitioners cannot be excluded. In Haiti, Somalia, and Afghanistan they were often the first point of contact for patients, particularly in rural regions. Information on the DRC is not as clear. Small scale studies imply that they do not play as big a role, this goes against reports from the Ministry of Health and what is known about the use of traditional medicine in other countries [31].

The establishment of private pharmacies and pharmaceutical vendors as sources of health care information can have a negative impact on the rational use of medicines because oftentimes information is biased, incorrect or simply not provided. But the entrenchment of the private pharmacy as an alternative to state pharmaceutical supply strongly suggests that it cannot be eliminated; reforms around it must be implemented carefully to work towards raising their quality bar in terms of patient services and health outcomes. Over-regulating the sector could drive it further underground, endangering the public health situation further, suggesting that cooperative regulation is needed. The public is not necessarily opposed to regulation, so long as it leads to a better provision of service, not worse. Lessons could be drawn from countries like Nigeria where patent medicine vendors have organized amongst themselves to maintain some control over the services provided [43]. Although there was insufficient communication between the government and the patent medicine vendors, existing associations of the vendors attempted to regulate seller actions.

### Rational medicines Use

Irrational use of medicines is a broad area encompassing inappropriate prescription practices, the sale of pharmaceuticals and how well patients follow their pharmaceutical regimen [44]. Rational use of medicines is affected by the quality of the health system, in terms of where patients go for care and where they fill out their prescriptions. Quality also includes the perceptions of the patients on the care they receive and the pharmaceuticals that are prescribed. Irrational medicines stem in part from the perception of low quality public health care systems as well as a weak understanding of the impact of improper use of medicines. On the other hand, the easily accessible and low priced pharmaceuticals sold in markets must also attract customers as a rational, price-sensitive appraisal of available options for care. Common practices include providing medicines without prescriptions, improper diagnoses due to lack of training and business considerations, and perceptions that more pills mean more improvement in health status. Consistent to this logic, ‘free’ medicines are deemed to be valueless. There is plenty of anecdotal evidence supporting the idea that families prefer buying expensive medicines that they believe, because of the supposed high value, are more powerful.

One of the prominent trends across the four countries was the fact that accessibility of public health care did not explain the large amount of drug sellers. The greatest concentration of sellers was usually found in urban areas, where there is likely a greater amount of public health facilities as well. One caveat to this observation is that there has been insufficient research to determine the distribution of drug sellers in rural regions. Nevertheless, it is clear that they are an important part of the healthcare sector in urban areas and access should not be excluded as a concern altogether. In Somalia, the long operating hours of non-public pharmacies was an attractive aspect for citizens. Therefore, while mobility may not be a major limiting factor, hours of operation can be. Several other factors likely direct citizens to drug sellers. Out of pocket expenditures form a large portion of health financing in each country, making cost a likely concern. Although their prices have not been examined, it is reasonable to believe that their need to compete amongst each other has resulted in lower prices. As well, as was the case in the DRC, citizens in any of the countries may believe that any pill represents curative care. Drug sellers regularly sell without prescriptions, and at times, may even take on the role of the prescriber without any of the necessary training.

Given the inherent danger and opportunity costs in the irrational use of medicines, this is an area where strategic interventions could be targeted. Implementing educational campaigns to highlight the risks involved in poor prescribing practices is one low cost intervention that could potentially alert more of the patients to potential risks. In addition to demand-side initiatives, supply-side incentives must be deployed.

### Counterfeits

The definition of a counterfeit medicine varies among countries but the most widely accepted definition is given by the WHO as a medicine.

“(W)hich is deliberately and fraudulently mislabeled with respect to identity and/or source. Counterfeiting can apply to both branded and generic products and counterfeit products may include products with the correct ingredients or with the wrong ingredients, without active ingredients, with insufficient active ingredients or with fake packaging” [45].

Counterfeit medicines fall under the classification of substandard pharmaceuticals, which are those manufactured below certain standards of quality and thus not therapeutically active. When the medicines are harmless in terms of toxicity, public health is still damaged as patients go untreated. Issues of counterfeit medicines are closely entwined with security concerns in severely disrupted countries due to the lack of the government’s ability to sufficiently monitor products going in and out of the country. As the quality assurance mechanisms necessary to manage substandard medicines in particular require significant resources and skills, it can be argued that there is a disincentive for pharmaceutical manufacturers to address the issue [46]. Substandard pharmaceuticals are not necessarily a product of counterfeit pharmaceutical manufacturing. Instead pharmaceuticals manufactured in legitimate factories are subject to different controls depending on the target country [46].

Maintaining quality in the pharmaceutical supply chain is a problem for all countries across both the private and public sectors. Each of the studied countries has had challenges in implementing regulations and especially in enforcing them. The porous borders in Afghanistan make it difficult to monitor imports, and though quality control policies are in place by law, they are not consistently enforced. In Haiti, the government has made an effort to begin monitoring imports but has met resistance from NGOs and other aid organizations because of resulting delays. Though this is a problem in emergency situations such as the cholera epidemic, according to informants it has also played a role in limiting dumping of medicines which are not needed. Most policies are in early stages, making it impossible to conclusively evaluate their strength. However, policy-makers should also keep in mind that these systems will never work perfectly, particularly in the regulation of informal sellers. Compromises will have to be made. The difference in quality between public, private and informal drug vendors varies by country, though often information is simply not available (as in the DRC). In Somalia, public hospitals would not purchase from market sellers. In Haiti, drugs being supplied through NGOs were not necessarily of optimal quality either, and at times they simply were not needed.

The area with the least amount of information available is unsurprisingly the trade of counterfeit medicines. It is evident that there is a significant counterfeit trade occurring both in Somalia and Afghanistan. Less is known about Haiti and the DRC, which definitely does not mean that it does not occur. For all of these countries, an attempt to map out the different sources of medicines outside the main supply channels is needed to allow for more informed decision-making when addressing the issue of counterfeits in both the public and private sectors.

Understanding the networks of the import and sale of counterfeit and sub-standard pharmaceuticals is integral to a deeper understanding of the informal markets. Better interventions can be developed which do not eliminate the informal market but make it safer for citizens. Importantly, wholesalers are the most common source of pharmaceuticals in the countries examined [23,37,13]. Though regulating quality at the point of sale may be difficult, targeting wholesalers could be considered as a feasible way to ensure better quality of pharmaceutical supplies in less than optimal pharmaceutical systems.

### Future directions

In our paper we have highlighted the importance of informal pharmaceutical supply systems in severely disrupted countries for people in need. In the absence of a functioning public sector, we found that informal supply systems necessarily thrive. While some of these systems work well enough to provide medicines to patients, others not surprisingly remain weak at best. Still, in the absence of formal supply, these systems provide some degree of pharmaceutical services to those in need, albeit they are far from perfect. Recognizing this reality, we propose a pragmatic approach to dealing with the informal pharmaceutical markets in states where public services are either non-existent or severely limited. We thus support that informal supplies of pharmaceutical systems need to be recognized and enhanced if we hope to make sustainable change to pharmaceutical supply to those in need. We indeed recognize the importance of supporting state capacity strengthening where it is feasible and realistic to do so, but simultaneously efforts should be directed to developing the informal sector as well. In other words, we support a top-down and bottom-up approach to pharmaceutical services.

Possible interventions should focus on monitoring current pharmaceutical supply strategies and tactics to identify areas where interventions are most relevant and likely to yield the biggest results in terms of public health and in improving the education of the population about the rational use of medicines. If patients continue to turn to the informal sector for their drug supply, education of these informal sellers is vital so they at least have a basic understanding of the product they are providing. Encouraging the use of prescriptions could also improve outcomes but would require closer integration of sellers with the public health system. The difficulty of coordinating the public sector itself suggests that it would be difficult to deepen the infrastructure to include the private sector. But any intervention must take stock of the motivations of the informal providers: without the right incentives, they will likely not adopt a desirable behavior, regardless of training [32]. For example if there is no competition for pharmaceutical supply, such as when a pharmaceutical seller has a monopoly in a remote village, prices can instead reflect what the market will allow for, and put additional financial burden on the poor.

We have focused on non-traditional methods of providing access to medicines to patients in need. We believe the informal market suppliers should be integrated into any existing initiatives that aim to improve access to medicines. While donor programmes can undeniably help fill in the gap, they are clearly not fixing the problem but rather allowing for temporary solutions to long-term problems. While it is desirable to advance the development of the “ideal” pharmaceutical system, these efforts may in fact result in very little gains in the context of a severely disrupted country. For instance, in Somalia, the privatization of the pharmaceutical supply system is both extensive and deep, so outside efforts directed to reviving a public purchasing and supply system would have a limited impact.

We work with the reality that members of the population will seek out pharmaceutical products from available sources, such as private pharmacies and informal vendors. It is evident that in the face of weak public healthcare systems, creative but unregulated options emerge in terms of pharmaceutical supply. These, however, may only represent interim coping mechanisms. Still, they need to be integrated when developing policy options for further interventions that aim to help improve the quality, consistency and access of the population to medicines.

Where private pharmacies are providing services beyond the sale of medicines, these can be scaled up to produce available health care services when public sector fail, but there is a need for the training of these pharmaceutical sellers even if they are not qualified health care professionals. This could include education on strategies for identifying substandard and counterfeit pharmaceuticals and basic information on medicine use. In the context of state break-down, traditional models are desirable but not realistic. We thus advance less than perfect, but realistic solutions to pharmaceutical supply for those in need.

## Competing interests

The authors have declared no competing interests.

## Authors' contributions

JCK developed research paper design and was the primary author. EP developed the research concept, completed field work for case studies, and took part in critical revision. M. Markus developed the research concept, completed field work for case studies and took part in critical revision. NO took part in analysis and interpretation of field research and helped draft the manuscript. M. Murru completed field work for case studies and took part in critical revision. PH completed field work for case studies and took part in critical revision. All authors read and approved the final manuscript.

## Pre-publication history

The pre-publication history for this paper can be accessed here:

http://www.biomedcentral.com/1472-698X/12/34/prepub

## References

[B1] Kohler J Fighting corruption in the health sector: methods, tools and good practices 2011 New York: UNDP

[B2] United Nations Development Programme Governance for sustainable human development: a UNDP policy document 1997 New York: United Nations Development Programme

[B3] OECD Service delivery in fragile situations: Key concepts, findings, and lessons 2008 Paris: OECD

[B4] Zoellick RB Fragile states: securing development Survival 2008 50 6 67 84 10.1080/00396330802601859

[B5] Carlson C de Lamalle JP Fustukian S Newell-Jones K Sibbons M Sondorp E Improving the delivery of health and education services in difficult environments: lessons from case studies 2005 London: DFID Health Systems Resource Centre

[B6] Briscoe I The EU response to fragile states European Secur Rev 2008 42 7 10

[B7] Meesen B Bigdeli M Chheng K Decoster K Ir P Men C Van Damme W Composition of pluralistic health systems: how much can we learn from household surveys? an exploration in Cambodia Health Policy Plan 2011 26 i30 i44 10.1093/heapol/czr026 21729915

[B8] Walle GV Ponsaers P Formal and informal pharmaceutical economies in third world countries: synergetic, symbiotic, or parasitical? Crime Law Soc Change 2006 45 361 372 10.1007/s10611-006-9039-z

[B9] Belay TA “Building on early gains in Afghanistan’s health, nutrition, and population sector: challenges and options 2010 Washington: The World Bank

[B10] Alsi P Amare H Boccanera R Intili JA Kissm E Khuram MD Williams S Afghanistan private sector health survey. Global health technical assistance project report No. 08-001-138 2009 Washington, DC: USAID

[B11] Harper J Strote G Afghanistan pharmaceutical sector development: problems and prospects Southern Med Rev 2011 4 1 29 39

[B12] Harper JR Shahab A Afghanistan pharmaceutical sector EC identification mission report (FWC contract 144147) 2008 European Commission

[B13] Paterson A Karimi A Understanding markets in Afghanistan: a study of the market for pharmaceuticals 2005 Kabul, Afghanistan: Afghanistan Research and Evaluation Unit

[B14] Ministry of Public Health / European Commission Health financing review 2008 Kabul, Afghanistan: Islamic Republic of Afghanistan Ministry of Public Health

[B15] Ministry of Public Health Monitoring & Evaluation Department, with Johns Hopkins University, Bloomberg School of Public Health and Indian Institute of Health Management Research Afghanistan health survey 2006: estimates of priority health indicators for rural Afghanistan general directorate of policy and planning 2006 Kabul, Afghanistan

[B16] Afghanistan Ministry of Public Health Public Nutrition Department Afghanistan national development strategy (ANDS): draft health and nutrition sector strategy 2008–2013 2008 Kabul, Afghanistan: Islamic Republic of Afghanistan Ministry of Public Health

[B17] Chenin B Report 6: The revolving drug fund. Bringing back the ‘public’ into the Somaliland public hospitals. Hargeisa group hospital, Somaliland 2008 Somalia: UNICEF and European Union Available online at: http://www.unicef.org/somalia/health_11679.html

[B18] Ali GKM Report on revolving drug fund evaluation and consultancy 2008 London, UK: Tropical Health and Education Trust

[B19] Jeene H The health system in karkaar region, puntland, Somalia 2010 London, UK: Save the Children UK

[B20] Mazzilli C Ahmed R Davis A Report 11: The Private Sector and Health: a survey of Somaliland private pharmacies 2009 Geneva: UNICEF and European Union Available online at: http://www.unicef.org/somalia/health_11705.html

[B21] Capobianco E Naidu V A decade of aid to the health sector in Somalia (2000–2009) 2010 Washington: The World Bank

[B22] Marchal R A survey of Mogadishu’s economy 2002 Nairobi: European Commission/Somali Unit

[B23] Kogi-Makau W Opiyo R Somali knowledge attitude and practices study. Infant and young child feeding and health seeking practices 2007 Nairobi, Kenya: Food Security Analysis Unit, Food and Agriculture Organization

[B24] Pan American Health Organization Haiti: Profile of the Health Services System 2003 Haiti: Pan American Health Organization

[B25] Delorme G Durand-Drouhin JL Vargas V Barry A Vandenplas B Day G Des Rosiers S Audit organisationnel – draft. Port-au-prince 2010 Haiti: Ministere de la Sante Publique et de la Population (MSPP)

[B26] Pierre Y-F Tardieu J-F Etude diagnostique sur la gouvernance de l’Hôpital Justinien du Cap-Haïtien et de l’Hôpital de l’Université de l’Etat d’Haïti; La Fondation Héritage pour Haïti, Le Centre pour l’Ethique et l’Intégrité Publique et Privée, Section haïtienne de Transparency International 2007

[B27] Petit-Frere I Joseph JM Henrys JH Le droit medical haitien – etat des lieux. Port-au-Prince: Unite de Recherche et d'Action Medico Legale 2009 Available at: http://www.uramel.org

[B28] Hatløy A Enquete sur les conditions de Vie en haïti. Port-au-prince 2001 Haiti: Institut Haitien de Statistique et d’Informatique

[B29] Ivers LC Garfein ES Augustin J Raymonville M Yang AT Sugarbaker DS Farmer P Increasing access to surgical services for the poor in rural haïti: surgery as a public good for public health World J Surg 2008 32 537 542 10.1007/s00268-008-9527-7 18320267 PMC2267856

[B30] Jones SG Hilborne LH Ross Anthony C Davis LM Girosi F Benard C Swanger RM Datar G Timilsina A Securing health: lessons from nation-building missions 2006 Santa Monica, CA: RAND Corporation

[B31] Bank W Democratic republic of Congo health, nutrition and population: country status report 2005 DC: Washington

[B32] Kassa A Access to healthcare, morality and violence in democratic republic of Congo 2005 Brussels: Medecins Sans Frontieres

[B33] Ministère de la Santé Publique de la République Démocratique du Congo Cartographie des systèmes d’approvisionnement et de distribution des médicaments et autres produits de santé en RDC 2010 Kinshasa, Democratic Republic of Congo: World Health Organization

[B34] BERCI Etat des lieux de mecanismes de gestion et de l’efficacite de la fourniture des services de base au niveau local en RDC. Kinshasa, democratic republic of Congo; 2004 World bank: democratic republic of Congo health, nutrition and population 2005 Washington, DC: Country Status Report

[B35] Putzel J Lindemann S Schouten C Drives of change in the democratic republic of Congo: the rise and decline of the state and challenges for reconstruction - crisis state working papers series N° 2 2008 London, UK: Crisis States Research Centre

[B36] Kizito Nsarhaza B The informal pharmaceuticals market in the democratic republic of Congo Dev Pract 1996 8 241 245

[B37] Bishikwabo KN The informal pharmaceuticals market in the democratic republic of Congo Dev Pract 1998 8 241 245 10.1080/09614529853882

[B38] Health Systems 20/20 Project Comptes nationaux de la santé 2008–2009 2011 Bethesda, MD: Health Systems 20/20 project, Abt Associates Inc

[B39] International Rescue Committee Emergency assistance to displaced and host communities in the rwanguba health zone, north Kivu, end of project evaluation 2009 Unpublished report

[B40] Persyn P Ladrière F Trefon T The miracle of life in Kinshasa: new approaches to public health Reinventing order in the Congo: How people respond to state failure in Kinshasa 2004 Kampala: Fountain Publishers and London Zed Books

[B41] Ministère de la Santé Publique de la République Démocratique du Congo Politique pharmaceutique nationale 2005 Brazzaville: Ministère de la Santé Publique de la République Démocratique du Congo

[B42] Doevenspeck M Mwanabiningo NM Bruns B, Miggelbrink J Navigating uncertainty: observations from the Congo-Rwanda border Subverting borders 2012 Wiesbaden: Springer

[B43] Oladepo O Salami KK Adeoye BW Oshiname F Ofi B Oladepo M Ogunbemi O Lawal A Brieger WR Bloom G Peters DH Malaria treatment and policy in three regions in Nigeria: the role of patent medicine vendors 2007 Future Health Systems No location provided. Available online at http://www.futurehealthsystems.org

[B44] World Health Organization - Rational Use of Medicines Accessed November 27, 2011. http://www.who.int/medicines/areas/rational_use/en/

[B45] World Health Organization General information on counterfeit medicines Accessed November 26, 2011. Available at http://www.who.int/medicines/services/counterfeit/overview/en/

[B46] Caudron JM Ford N Henkens M Mace C Kiddelle-Monroe R Pinel J Substandard medicines in resource-poor settings: a problem that can no longer be ignored Trop Med Int Health 2008 13 8 1062 1072 10.1111/j.1365-3156.2008.02106.x 18631318

